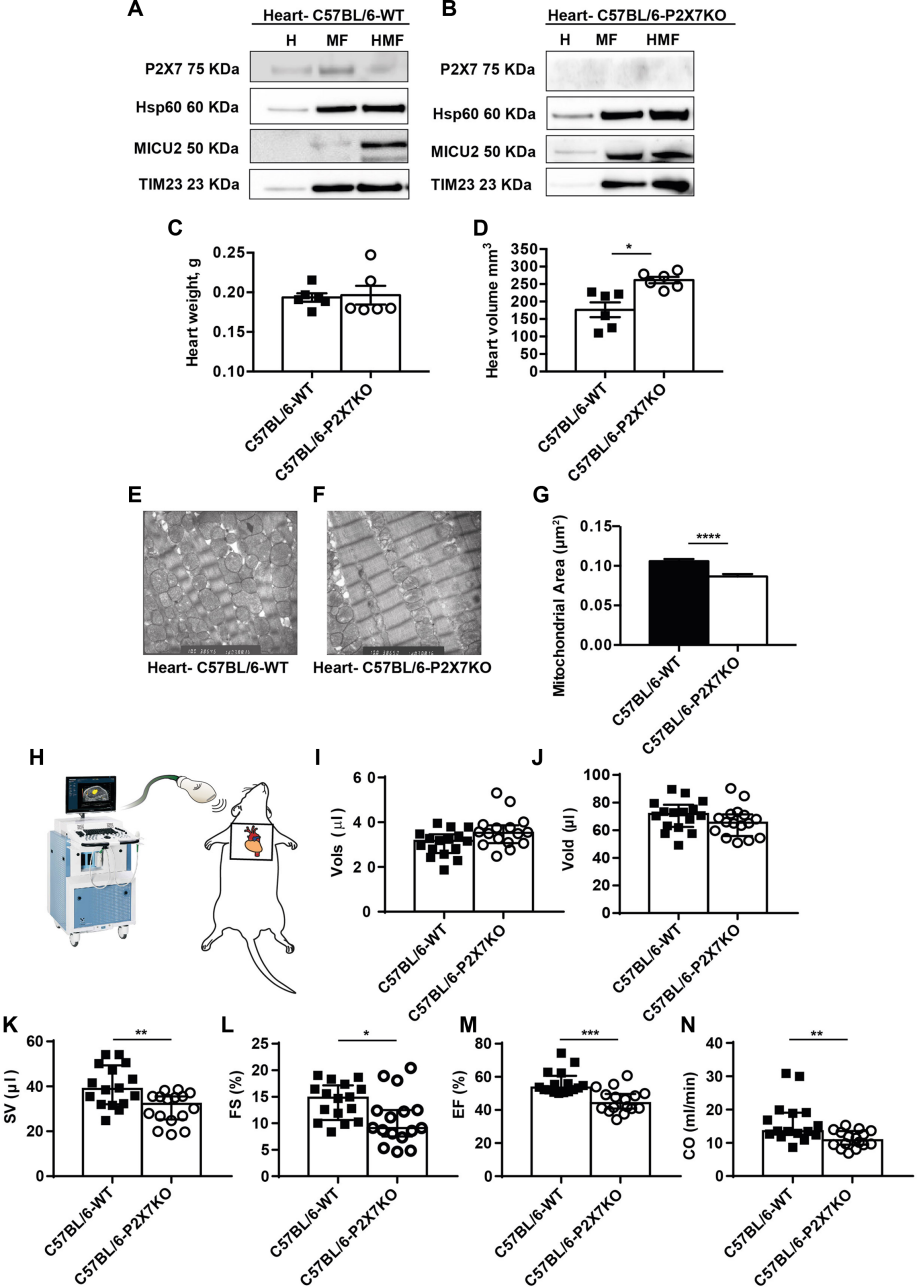# Corrigendum to Sarti et al. Mitochondrial P2X7 Receptor Localization Modulates Energy Metabolism Enhancing Physical Performance

**DOI:** 10.1093/function/zqab025

**Published:** 2021-06-18

Address correspondence to Francesco Di Virgilio (e-mail: fdv@unife.it)

In Panel H of Figure 1, the colors of the bars for HEK293 and HEK293-P2 x 7 cells were inverted. This has been amended. The corrected Figure 1 is published within this notice.

**Figure 1.**
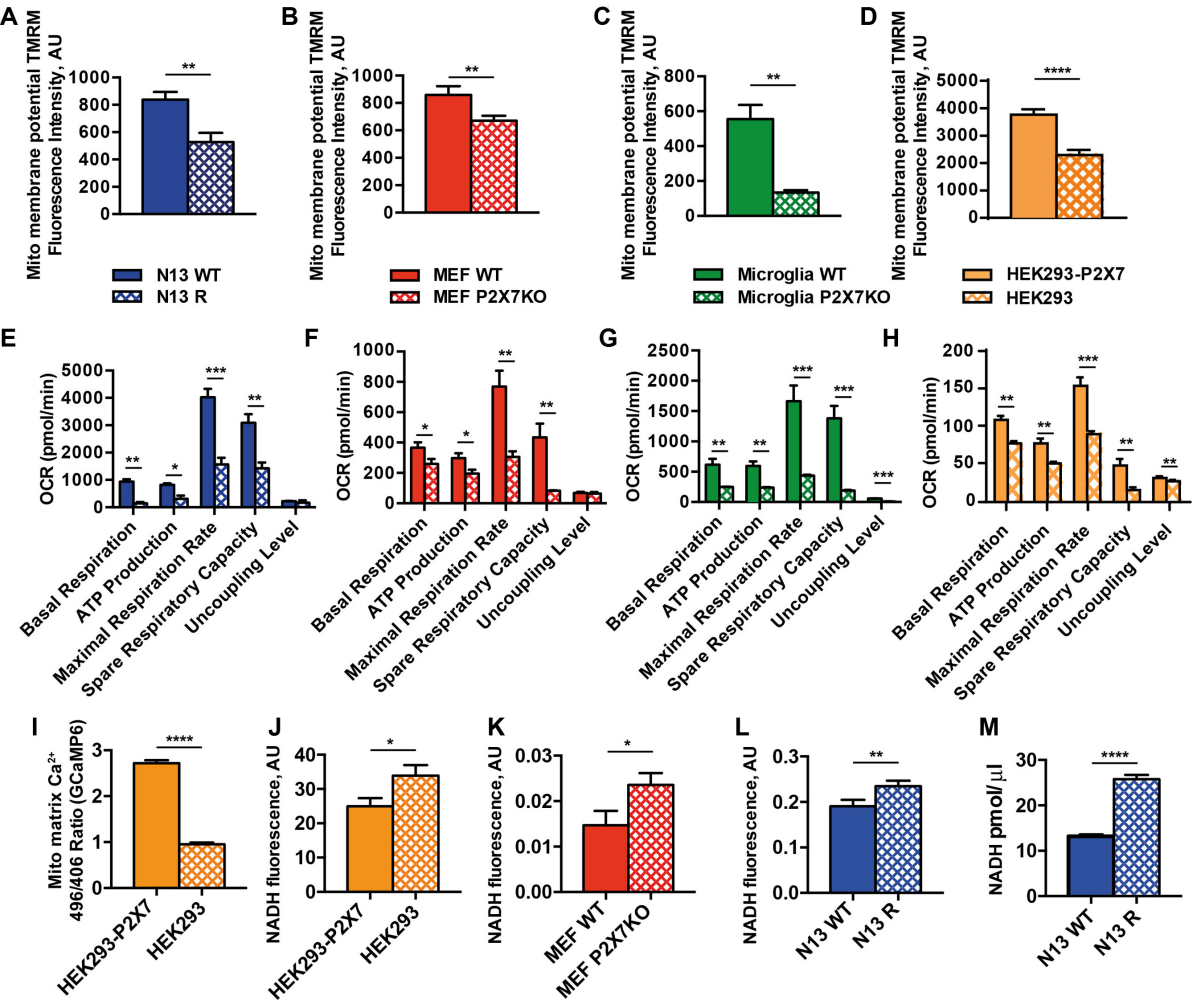


In Figure 6, Panel L and Panel M have been amended to display Fractional Shorting (FS) and Ejection Fraction (EF) as percentages. The corrected Figure 6 is published within this notice.

**Figure 6.**