# Supplementary material for: Mitochondrial P2X7 Receptor Localization Modulates Energy Metabolism Enhancing Physical Performance
Source: Function (Oxf). 2021 Jan 28;2(2):zqab005. doi: 10.1093/function/zqab005 (PMC8788778; doi:10.1093/function/zqab005)
Supplement: zqab005_Supplementary_Data [file zqab005_supplementary_data.docx]

**Table S1**

|  | MEF WT | MEF P2X7-KO |
| --- | --- | --- |
| Basal Respiration | 376 ± 25.77 N=3 | 258.7 ± 33.33 N=3 |
| ATP Production | 302.7 ± 28.10 N=3 | 195 ± 25.94 N=3 |
| Maximal Respiratory Rate | 769.3 ± 104.9 N=3 | 305.7 ± 37.17 N=3 |
| Spare Respiratory Capacity | 434.7 ± 89.89 N=3 | 82.67 ± 3.180 N=3 |
| Uncoupling Level | 68.67 ± 7.172 N=3 | 63.67 ± 10.84 N=3 |

|  | N13 WT | N13 R |
| --- | --- | --- |
| Basal Respiration | 938.7 ± 82.60 N=3 | 143.3 ± 48.46 N=3 |
| ATP Production | 816.7 ± 59.89 N=3 | 313 ± 114.6 N=3 |
| Maximal Respiratory Rate | 4025 ± 300.9 N=3 | 1565 ± 243.7 N=3 |
| Spare Respiratory Capacity | 3087 ± 318.2 N=3 | 1421 ± 212.8 N=3 |
| Uncoupling Level | 221.3 ± 13.87 N=3 | 171 ± 84.54 N=3 |

|  | Microglia WT | Microglia P2X7KO |
| --- | --- | --- |
| Basal Respiration | 616.3 ± 96.67 N=3 | 248.3 ± 4.096 N=3 |
| ATP Production | 597 ± 72.11 N=3 | 239 ± 7.234 N=3 |
| Maximal Respiratory Rate | 1663 ± 257.5 N=3 | 435.3 ± 14.62 N=3 |
| Spare Respiratory Capacity | 1381 ± 118.6 N=3 | 188 ± 10.26 N=3 |
| Uncoupling Level | 57 ± 1.155 N=3 | 9.333 ± 3.383 N=3 |

|  | HEK293-P2X7 | HEK293 |
| --- | --- | --- |
| Basal Respiration | 107.9 ± 5.974 N=6 | 78.57 ± 3.205 N=7 |
| ATP Production | 73.81 ± 5.487 N=6 | 51.30 ± 2.350 N=7 |
| Maximal Respiratory Rate | 156.7 ± 11.76 N=6 | 93.69 ± 3.845 N=7 |
| Spare Respiratory Capacity | 48.78 ± 8.730 N=6 | 15.12 ± 4.040 N=7 |
| Uncoupling Level | 34.10 ± 0.8806 N=6 | 27.27 ± 1.630 N=7 |

**Table S2**

|  | CTRL | BzATP |
| --- | --- | --- |
| CTRL vs. BZATP 10 min | 0.7487 ± 0.02080 N=7 | 0.8478 ± 0.01876 N=10 |
| CTRL vs. BZATP 1 H | 0.7487 ± 0.02080 N=7 | 0.9293 ± 0.02230 N=8 |
| CTRL vs. BZATP 6H | 0.7487 ± 0.02080 N=7 | 0.8801 ± 0.02092 N=11 |

|  | CTRL | Rotenone |
| --- | --- | --- |
| CTRL vs. Rotenone 10 min | 0.7487 ± 0.02080 N=7 | 0.7877 ± 0.02371 N=7 |
| CTRL vs. Rotenone 1 H | 0.7487 ± 0.02080 N=7 | 0.8836 ± 0.008011 N=7 |
| CTRL vs. Rotenone 6H | 0.7487 ± 0.02080 N=7 | 0.7639 ± 0.05414 N=7 |

|  | CTRL | H_2_O_2_ |
| --- | --- | --- |
| CTRL vs. H_2_O_2_ 10 min | 0.7487 ± 0.02080 N=7 | 0.8720 ± 0.01481 N=8 |
| CTRL vs. H_2_O_2_ 1 H | 0.7487 ± 0.02080 N=7 | 0.7936 ± 0.02162 N=7 |
| CTRL vs. H_2_O_2_ 6H | 0.7487 ± 0.02080 N=7 | 0.8716 ± 0.01225 N=7 |

.
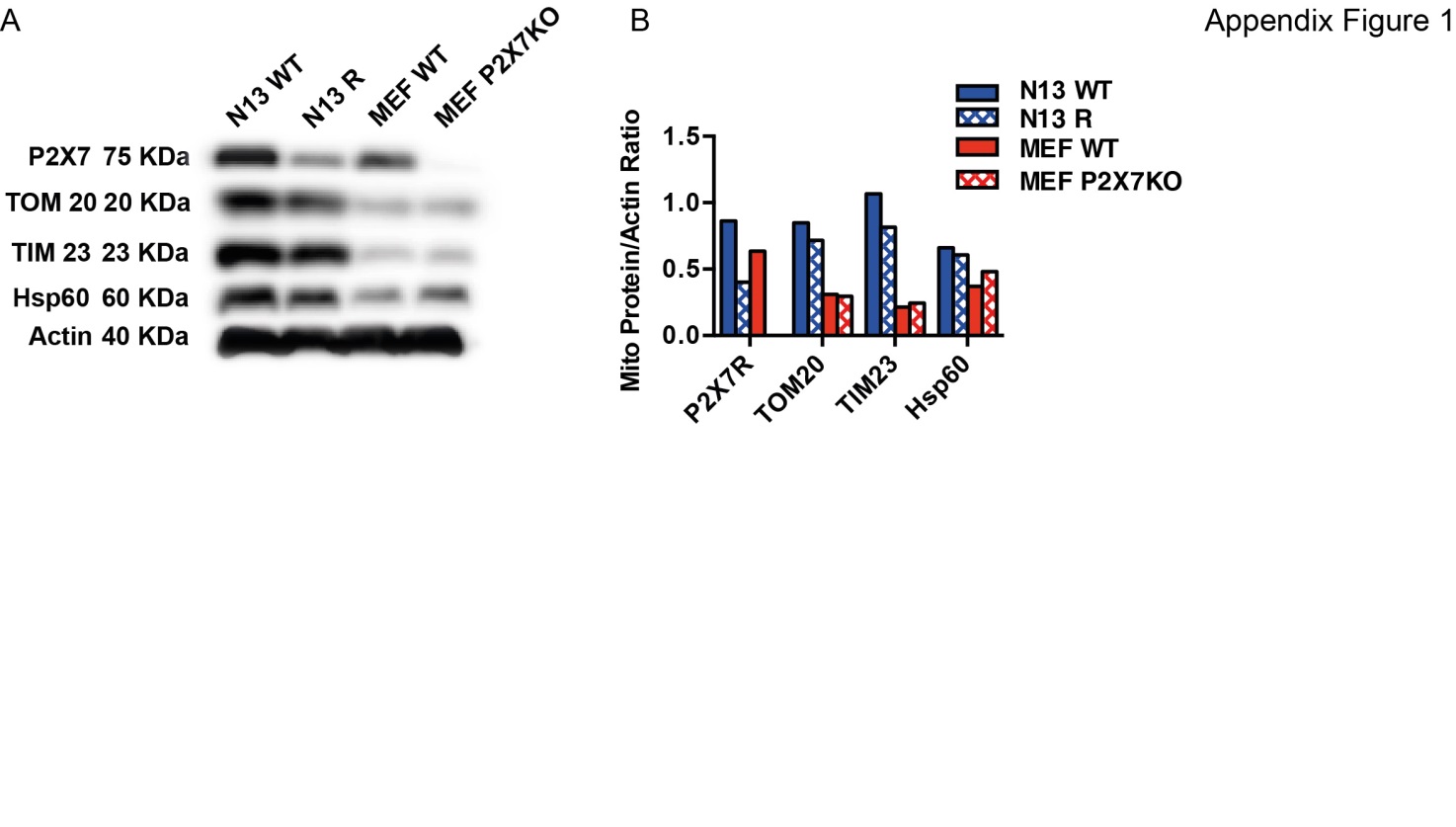


**Appendix Figure 1**

Lack of the P2X7R does not affect mitochondrial cell content.

Western blot analysis (A) and densitometry (B) of TOM20, TIM23 and Hsp60 content of N13 WT or N13 R, and MEF WT or MEF P2X7-KO cells.
